# Neoadjuvant intensity modulated radiotherapy for a single and small (≤5 cm) hepatitis B virus-related hepatocellular carcinoma predicted to have high risks of microvascular invasion: a randomized clinical trial

**DOI:** 10.1097/JS9.0000000000000574

**Published:** 2023-06-22

**Authors:** Xubiao Wei, Yabo Jiang, Shuang Feng, Chongde Lu, Lei Huo, Bin Zhou, Yan Meng, Wan Yee Lau, Yaxin Zheng, Shuqun Cheng

**Affiliations:** Departments of a Hepatic Surgery VI; b Radiotherapy; c Department of Radiology, Eastern Hepatobiliary Surgery Hospital, Navy Medical University, Shanghai; d Faculty of Medicine, The Chinese University of Hong Kong, Shatin, Hong Kong SAR, China

**Keywords:** hepatectomy, hepatocellular carcinoma, microvascular invasion, neoadjuvant radiotherapy, tumour recurrence

## Abstract

**Background:**

The presence of microvascular invasion (MVI) significantly impairs postoperative long-term survival of patients with hepatocellular carcinoma (HCC). The role of neoadjuvant radiotherapy (RT) in treating patients with an early-stage HCC predicted to have high risks of MVI remains to be explored.

**Materials and methods:**

Consecutive patients with a resectable single and small (≤5 cm) hepatitis B virus-related HCC predicted to have high risks of MVI were randomized 1:1 to receive either neoadjuvant intensity modulated radiation therapy (18 Gy with fractionated doses of 3 Gy) followed by surgery 4 weeks later or upfront surgery. The primary endpoint was disease-free survival (DFS). The secondary outcomes included overall survival (OS), objective response rate, RT-related toxicity and surgical complications.

**Results:**

There were 30 patients randomized to each of the two groups. In the neoadjuvant RT group, three patients violated the study protocol, with two having upfront hepatectomy and one radiofrequency ablation after RT. The objective response rate after RT was 25.0% (7/28), but 2 patients suffered from grade 3 liver toxicity. The median follow-up was 68 months (interquartile range, 58–70 months) in the neoadjuvant RT group, and 68 months (interquartile range, 62–75 months) in the upfront surgery group. On intention-to-treat analysis, the median DFS and median OS were not reached in both the 2 arms. The 1-year, 2-year, 3-year and 5-year DFS rates for the neoadjuvant RT group were 86.7%, 76.7%, 60.0% and 56.3%, versus 90.0%, 66.7%, 52.8% and 45.7% in the upfront surgery group (*P*=0.448), respectively. The corresponding OS rates were 96.7%, 86.7%, 83.3% and 72.7%, versus 100.0%, 93.3%, 79.6% and 60.7% (*P* = 0.399).

**Conclusion and relevance:**

For patients with a resectable single and small hepatitis B virus-related HCC predicted to have high risks of MVI, neoadjuvant RT gave a promising response rate with a mild toxicity. Nevertheless, the neoadjuvant RT yielded similar long-term DFS and OS rates compared with patients who underwent upfront surgery.

## Introduction

Highlights

- Our study examines the impact of microvascular invasion (MVI) on the postoperative survival of patients with hepatitis B virus-related Barcelona Clinic Liver Cancer stage A hepatocellular carcinoma (HCC). MVI’s presence significantly affects these patients’ survival outcomes. While neoadjuvant radiotherapy has been established as a beneficial treatment in reducing recurrence rates and improving postoperative survival in selected patients with resectable HCC and major vascular invasion, its potential benefits for patients with early-stage hepatitis B virus-related Barcelona Clinic Liver Cancer HCC and a high risk of MVI is yet to be clearly understood.
- In this randomized clinical trial, 60 patients were evaluated. The disease-free survival rates for the neoadjuvant radiotherapy group at 1, 2, 3 and 5 years were 86.7%, 76.7%, 60.0% and 56.3% respectively. In contrast, the survival rates in the surgery-alone group were 90.0%, 66.7%, 52.8% and 45.7% respectively (*P*=0.448) in the intention-to-treat population.
- Our findings suggest that there is no statistically significant difference in survival outcomes between neoadjuvant radiotherapy and upfront surgery for patients with early-stage HCC and a high risk of MVI. Therefore, the role of neoadjuvant radiotherapy in this specific patient population warrants further investigation.

In 2020, primary liver malignancy was the sixth most common cancer and the second leading cause of cancer-related deaths worldwide^1^. Hepatocellular carcinoma (HCC) accounts for ~75% of all primary liver cancers^2^. Hepatitis B virus (HBV) infection accounts for more than 60% of HCC cases in Asia and Africa, especially in China^3^. Liver resection is a potentially curative treatment which can offer favourable prognosis for early-stage HCC with a single lesion^4^. However, presence of microscopic vascular invasion (MVI) defined as microscopically identified cancer cell nests in portal or hepatic veins in adjacent liver tissues contiguous to the tumour indicates aggressive behaviour of HCC and compromises long-term post-treatment survival of these patients, with a significantly reduced disease-free survival (DFS) at 3 years (relative risk = 1.82)^5–7^. Our previous studies suggested that neoadjuvant radiotherapy (RT) prolonged overall survival (OS) and DFS in selected patients with resectable HCC and macrovascular invasion^8,9^. The aim of this prospective randomized controlled trial was to explore whether neoadjuvant RT could provide survival benefits to patients with a single and small (≤5 cm) HBV-related HCC predicted to have high risks of MVI.

## Methods

### Study design and participants

This randomized, open-label, controlled clinical study was approved by the Ethics Committee of our institute, and its protocol conformed to the standards of the 1975 Declaration of Helsinki. All patients provided written informed consent before participation. The trial also conformed to the 2010 CONSORT guidelines^10^, Supplemental Digital Content 1, http://links.lww.com/JS9/A731 and was registered at the Chinese Clinical Trial Registry.

The inclusion criteria were patients with: (1) age ranging from 20 to 70 years; (2) HCC diagnosed by biopsy or by the noninvasive criteria according to the European Association for the Study of Liver guidelines^11^. (3) hepatitis B surface antigen positivity; (4) a solitary tumour with a maximum diameter less than or equal to 5 cm and was assessed to be resectable using the Criteria in the Appendix, Supplemental Digital Content 2, http://links.lww.com/JS9/A732; (5) preoperative clinical parameters in predicting a high risk of MVI presence on subsequent histopathological study, based on the nomogram developed by Lei *et al* ^12^. This nomogram used seven independent factors to predict presence of MVI with a positive predictive value of 57.9% and a negative predictive value of 83.2%. In this study, we calculated the total score for predicting the risk of MVI using all seven predictive factors from the original nomogram, but with ‘tumour number’ contributing a fixed score of ‘0’ for all patients due to their having a single tumour. The other six factors, including large tumour diameter, incomplete tumour encapsulation on medical imaging, α-fetoprotein level greater than or equal to 20 ng/ml, platelet count less than 100 × 10^3^/µl, HBV DNA load greater than 10^4^ IU/ml, and the presence of the typical ‘arterial enhancement and washout’ sign on contrast-enhanced MRI, provided variability in the total scores. Patients with a nomogram score greater than or equal to 200 were considered to have high risks of MVI presence and were included in this study.

The exclusion criteria were patients with: (1) a history of other malignancy in the past five years; (2) any previous antitumour treatment for HCC within 1 year; and (3) hepatitis C virus or HIV co-infection.

### Sample size estimation

Based on a previous report^13^ and the results of subgroup analysis of a large cohort of patients treated from 2003 to 2013 at our institution^14^, the 5-year DFS rate was estimated to be 40% in patients who had a single and small (≤5 cm) HCC predicted to have high risks of MVI based on Lei’s criteria^12^, and 70% in patients who had no MVI on histopathological examination of resected specimens after surgery^15^. It was also assumed that, after neoadjuvant RT, patients initially at high risk of MVI would achieve a 5-year DFS rate equivalent to that of patients who received upfront surgery and showed no histopathologically identified MVI. Using a two-sided test with 80% power and a significance level of 5%, the minimum sample size was 27 patients for each of the two groups. With 10% of patients being added to allow for subsequent loss to follow-up, 30 patients were determined to be included into each of the two groups.

### Randomization

Eligible patients were assigned in a 1:1 ratio to either the neoadjuvant RT or the upfront surgery groups based on a computer-generated randomization code for treatment group allocations without stratification. The allocation was blinded to the researchers who performed the data analyses, but it was not blinded to the patients and clinicians.

### Interventions

For patients in the neoadjuvant RT group, intensity modulated radiation therapy (IMRT) was carried out within 1 week after randomization. The gross tumour volume was defined as the tumour volume that was enhanced in the arterial phase. The clinical tumour volume was generated by adding 5–10 mm around the tumour as calculated for the gross tumour volume. The planning target volume was expanded to include a 5–10 mm margin, to compensate for any internal physiologic movements and variations in size, shape and position of clinical tumour volume. The planned total radiation dose for the planning target volume was 18 Gy, delivered in fractions of 3.0 Gy using 6MV X-rays with a linear accelerator (Elekta Synergy), at a rate of 5 fractions per week. Before each treatment session, patients underwent a megavoltage cone-beam computed tomography and were positioned with automated image registration by application of the image guided RT system (Elekta Synergy). The respiratory gating technique was used to reduce the dose delivered to healthy tissues and surrounding organs. Patients undergoing IMRT were put in a supine position with arms above the head and they were instructed to breathe quietly. The RT regimen was in accordance with that used in our previous studies^8,9^.

During the course of RT, in case of serious adverse events, which is defined as greater than or equal to grade 3 according to Common Terminology Criteria for Adverse Events (CTCAE) version 5.0^16^, treatment may be delayed for a maximum of 7 days. If symptoms persisted, further RT or the planned operation was terminated, and patients were recommended to receive other therapies.

For patients in the upfront surgery group, liver resection was carried out within 1 week after randomization. Patients assigned to the neoadjuvant RT group were re-evaluated 4 weeks after completion of RT. If no contraindications were found, hepatectomy was carried out within 1 week. Hepatectomy was performed either anatomically or non-anatomically, based on the liver functional reserve and the possibility of achieving a surgical margin greater than or equal to 1 cm to reduce the possibility of residual MVI at the liver transection plane^17^. Details of the surgical procedure is described in the Appendix. Histopathological examinations were carried out by two independent pathologists using the diagnostic criterion of MVI based on that reported by Roayaie *et al* ^18^. The number and spatial distribution of invaded vessels were utilized to stratify patients with MVI into two groups: the mild MVI group (M1), which comprised patients with 1–5 involved vessels confined within a 1 cm radius from the tumour margin, and the severe MVI group (M2), which included patients with either more than 5 invaded vessels or invaded vessels situated at a distance exceeding 1 cm from the tumour margin^19^. No adjuvant therapy was performed for all enroled patients after surgery, unless an R0 resection had not been performed.

### Assessment of outcomes and follow-up

The primary endpoint was DFS, which was defined as the time interval between the randomization to the time disease recurrence was first diagnosed or death from any cause. OS as one of the secondary outcomes was defined as the time from the randomization to death from any cause. Survival outcomes were analyzed in both the intention-to-treat (ITT) and per-protocol (PP) populations. For objective response rate which was used as the other secondary endpoint, it was based on the modified Response Evaluation Criteria in Solid Tumors (mRECIST; version 1.1). RT-related toxicity was evaluated based on the CTCAE version 5.0^16^. Surgery-related complication was defined as occurrence of any postoperative complication, and graded by the Clavien–Dindo Classification^20^. The protocol of follow-up is described in the Appendix.

### Statistical analysis

Variables were compared using the χ^2^ test, Fisher’s exact test, independent *t*-test, Mann–Whitney U test or Wilcoxon rank sum test, as appropriate. Survival curves for time-to-event variables were determined by the Kaplan–Meier method, and the log-rank test was used for treatment comparisons. The hazard ratio for survival and its 95% CI were calculated using the Cox proportional hazards regression model. Subgroup analyses on the effect of neoadjuvant RT were performed using clinicopathological variables to include age, sex, serum HBV DNA load, albumin-bilirubin grade, α-fetoprotein level, tumour size, as well as the parameters of hepatectomy and pathological examinations. A value of *P* less than 0.05 was considered statistically significant. All statistical analyses were performed with the Stata 17.0 software (StataCorp).

## Results

### Patient characteristics

**Figure 1:**
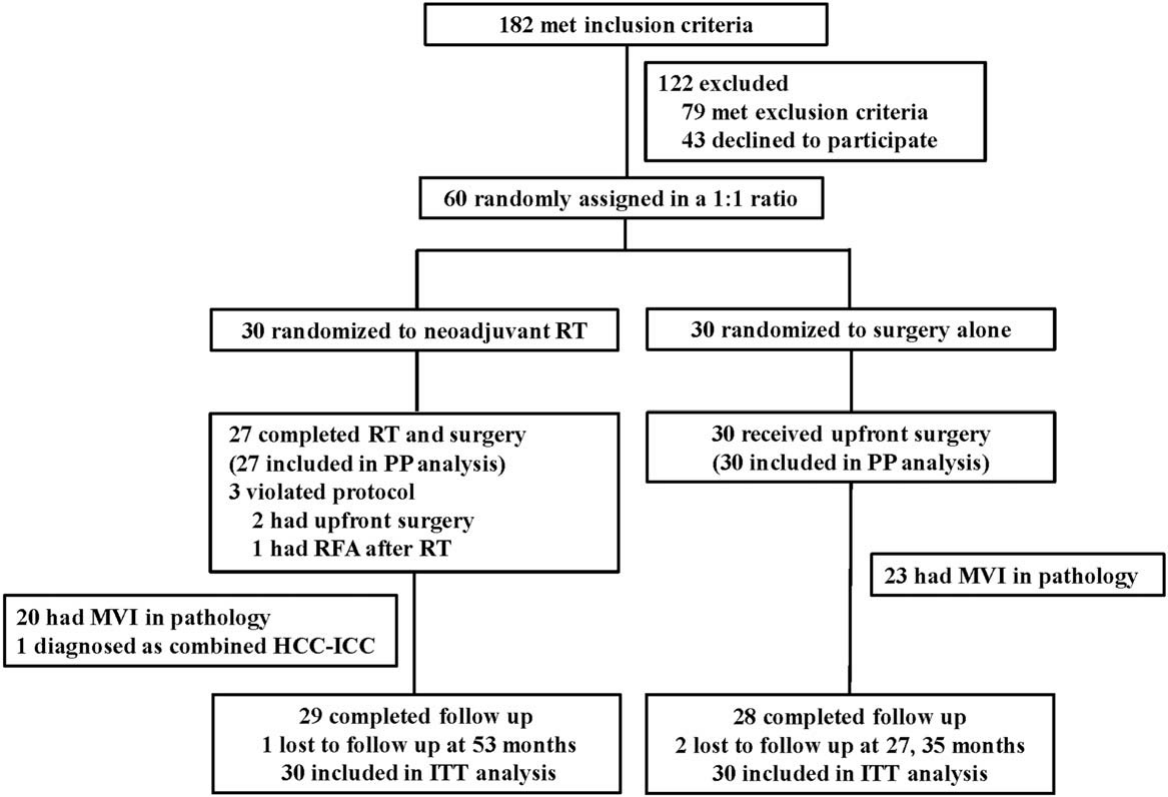
CONSORT diagram of the randomized clinical trial. HCC, hepatocellular carcinoma; ICC, intrahepatic cholangiocarcinoma; ITT, intention-to-treat; MVI, microvascular invasion; PP, per-protocol; RFA, radiofreqency ablation; RT, radiotherapy.

From October 2014 to January 2017, of 182 patients who were treated and assessed for eligibility to be included in this study, 122 were excluded either because of not meeting the inclusion criteria (*n* = 79) or refusing to participate (*n* = 43). Finally, 60 patients were randomly assigned in a 1:1 ratio to the neoadjuvant RT group (*n* = 30) and the upfront surgery group (*n* = 30; Fig. 1). In the neoadjuvant RT group, three patients violated the study protocol after randomization, with two having upfront hepatectomy and one having radiofrequency ablation after RT. These three patients were also analyzed in the neoadjuvant RT group according to the intention-to-treat principle.

The median scores of the MVI-predicating nomogram were 230 (interquartile range, 219–245) in the neoadjuvant RT group and 232.5 (interquartile range, 219–245) in the upfront surgery group (*P* = 0.619). Liver cirrhosis was confirmed histopathologically in 63.3% (38/60) of all the included patients, while comparison of liver function status showed no significant difference between the two groups, with the majority of patients having a Child-Pugh score of 5–6 (93.3%) and an albumin-bilirubin grade of 2 (68.3%). The proportions of patients had received anti-viral treatment, defined as a standard course of interferon therapy or the oral administration of anti-viral drugs for a duration of more than 2 months before randomization, were comparable between groups (*P* = 0.605). (Table 1) In this study, to prevent possible HBV reactivation during treatment, all patients with detectable HBV DNA level (>50 IU/ml) not currently undergoing anti-viral therapy were administered with a daily dose of 0.5 mg of entecavir.

**Table 1:** Comparison of clinicopathological characteristics between patients in neoadjuvant radiotherapy group and surgery-alone group.

Characteristics	Neoadjuvant RT (*n*=30)	Surgery alone (*n*=30)	*P*
General status			
Age (years, median), range	49 (30–70)	52 (36–70)	0.691
Sex (male)	22	23	0.889
ECOG score			
0	20	17	0.520
1	5	8	
Parameters of MVI nomogram			
Seral HBV DNA load (≥10 000 IU/ml)	13	15	0.605
AFP level (ng/ml)			0.109
≥400	18	25	
20–400	7	4	
≤20	5	1	
Tumour diameter (cm, median, interquartile range)	4.5 (4.0–4.8)	4.3 (4.0–4.8)	0.947
Platelet count (≤10^3^/ul )	12	7	0.165
Arterial enhancement in radiology	30	27	0.119
Incomplete capsule in radiology	26	24	0.365
Score of MVI nomogram (median, interquartile range)	230, 219–245	232.5, 220–255	0.619
Other baseline clinical parameters			
Previous antivirus treatment	9	13	0.284
Presence of liver cirrhosis	18	20	0.771
Child-pugh score			
5–6	28	28	1.0
7	2	2	
ALBI score			
1	10	9	0.781
2	20	21	
Data of surgical procedure^a^			
Type of hepatectomy			
Major	17	16	0.683
Minor	12	14	
Anatomical resection	11	9	0.520
Operating time (≥180 min)	9	12	0.472
Volume of blood loss (≥400 ml)	6	7	0.807
Intraoperative blood transfusion	2	3	1.0
Postoperative complication^b^			0.357
None	23	27	
Grade I/II	5	3	
Grade III/IV	1	0	
Pathological findings^a^			
Microvascular invasion			0.332
Severe (M2)	11	8	
Mild (M1)	9	15	
No (M0)	9	7	
Tumour differentiation			0.333
III/IV	25	22	
I/II	4	8	
Tumour capsule			0.360
Incomplete/absence	24	21	
Complete	5	9	
Resection margin			0.552
<1 cm	8	6	
≥1 cm	21	24	

a Pathological data were available in 29 patients in the neoadjuvant radiotherapy group with 1 had radiofrequence ablation after radiotherapy.

b Graded by Clavien–Dindo Classification.

Continous parameters were calculated using Mann–Whitney U test.

AFP, α-fetoprotein; ALBI, albumin-bilirubin; ECOG: Eastern cooperative oncology group; HBV, hepatitis B virus; MVI, microvacular invasion; RT, radiotherapy.

### Response rate and adverse events of radiotherapy

The objective response rate of RT was 25.0% (7 of 28 patients), with no complete response and progressive disease; seven (25.0%) patients had partial remission and 21 (75.0%) had stable disease. Most of the adverse events after RT were mild and required no treatment. The proportions of patients with greater than or equal to grade 3 adverse events in the neoadjuvant RT group are shown in Table 2. After RT, two patients suffered from grade 3 liver toxicity (two had elevated liver enzymes and one had elevated serum bilirubin) and four patients had grade 3–4 thrombocytopenia. Because of drug treatments of adverse events and for laboratory abnormalities, liver resection was delayed in five patients for 5–7 days. No patients had cancellation of surgery due to the toxicity of RT.

**Table 2:** Tumour response and adverse events in neoadjuvant radiotherapy group^a^.

Radiological response^a^	No. patients according to mRECIST, *n* (%)		
Complete response	0		
Partial response	7 (25.0)		
Stable disease	21 (75.0)		
Progressive disease	0		
	No. patients according to CTCAE grade, *n* (%)		
Adverse events	3	4	5
Fatigue	2 (7.1)	0	—
Anorexia	1 (3.6)	0	—
Bilirubin increased	1 (3.6)	0	—
ALT/AST increased	2 (7.1)	0	—
Alkaline phosphatase increased	1 (3.6)	0	—
Platelet count decreased^b^	3 (10.7)	1 (3.6)	—

a Radiotherapy response and safety analyzed in 28 patients who received neoadjuvant radiotherapy.

b Patients had decreased platelet count at initial evaluation but no significant decrease (≥20%) after radiotherapy were excluded.

ALT, alanine transaminase; AST, aspartate aminotransferase; CTCAE, Common Terminology Criteria for Adverse Event; mRECIST, modified Response Evaluation Criteria in Solid Tumors.

### Surgical and pathological information

The proportions of patients who underwent major hepatectomy and anatomical resection, the operation time, volume of blood loss, as well as the probability of intraoperative blood transfusion were comparable between the two groups. Major postoperative complications (Clavien–Dindo classification of grade 3 or above) were observed in one patient in the neoadjuvant RT group. There was no surgery-related mortality in both groups. In the neoadjuvant RT group, the proportion of patients with MVI as confirmed by histopathological examination was 69.0% (20/29, M1: 9, M2:11), compared with 76.7% (23/30, M1: 15, M2: 8) in the upfront surgery group (*P* = 0.506). (Table 1).

### Follow-up data

The median follow-up was 68 months (interquartile range, 58–70 months) for the neoadjuvant RT group and 68 months (interquartile range, 62–75 months) for the upfront surgery group. At the maximum follow-up of 84 months, 13 patients had developed tumour recurrence and 8 patients had died of HCC in the neoadjuvant RT group, compared with 16 patients who had developed recurrence and 11 who had died of tumour progression in the upfront surgery group. We compared the survival probabilities of patients with severe MVI (M2), mild MVI (M1) and no MVI (M0) using the Cox proportional hazards model. The analysis revealed a hierarchical structure in DFS (*P* = 0.067) and OS (*P* = 0.057), with M0 exhibiting the highest survival probability, followed by M1, and then M2, which had the lowest survival probability. (Supplemental Figure A1, Supplemental Digital Content 3, http://links.lww.com/JS9/A733) Patients who received neoadjuvant RT had a higher probability of developing multiple intrahepatic recurrences and distant metastasis versus patients who had upfront surgery (*P* = 0.041). The details of recurrences are listed in Supplemental Table A1, Supplemental Digital Content 4, http://links.lww.com/JS9/A734. Therapies of recurrences are listed in Supplemental Table A2, Supplemental Digital Content 4, http://links.lww.com/JS9/A734.

**Figure 2:**
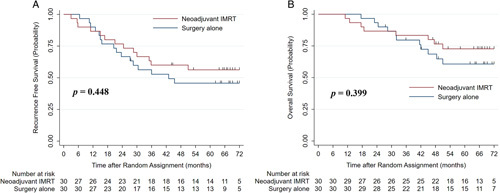
Disease-free (A) and overall (B) survival curves for the intensity modulated radiation therapy followed by surgery and upfront surgery groups in the intention-to-treat population. IMRT, intensity modulated radiation therapy.

In the ITT population, the median DFS and median OS were not reached in either of the two arms. The 1-year, 2-year, 3-year and 5-year DFS rates for the neoadjuvant RT group were 86.7%, 76.7%, 60.0% and 56.3%, versus 90.0%, 66.7%, 52.8% and 45.7% for the upfront surgery group (*P* = 0.448). (Fig. 2A) The 1-year, 2-year, 3-year and 5-year OS rates for the neoadjuvant RT group were 96.7%, 86.7%, 83.3% and 72.7%, versus 100.0%, 93.3%, 79.6% and 60.7% for the upfront surgery group. (*P* = 0.399) (Fig. 2B).

Specifically, the 3-year DFS rate was 60.0% (95% CI, 40.5–75.0%) in the neoadjuvant RT group versus 52.8% (95% CI, 33.6–68.8%) in the surgery-alone group (*P* = 0.578), and the corresponding 5-year DFS rate was 56.3% (95% CI, 36.8–71.8%) versus 45.7% (95% CI, 27.4–62.4%) (*P* = 0.446). Furthermore, the 3-year OS rate in the neoadjuvant RT group was 83.3% (95% CI, 64.5–92.7%) compared with 79.6% (95% CI, 60.1–90.3%) in the surgery-alone group (*P* = 0.798), and the corresponding 5-year OS rate was 72.7% (95% CI, 52.6–85.3%) versus 60.7% (95% CI, 40.2–76.1%) (*P* = 0.394). In the early recurrence group (≤ 2 years), the recurrence rate was 23.3% (7/30) in the RT group and 33.3% (10/30) in the surgery-alone group (*P* = 0.567). For the late recurrence group (> 2 years), the recurrence rate was 20.0% (6/30) in both the neoadjuvant RT group and the surgery-alone group (*P* = 1.0). (Supplemental Table A1, Supplemental Digital Content 4, http://links.lww.com/JS9/A734).

**Figure 3:**
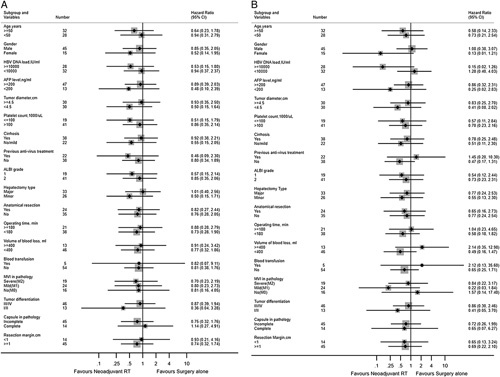
Forest plots of disease-free (A) and overall (B) survival in patient subgroups. AFP, α-fetoprotein; ALBI grade, albumin-bilirubin grade; HBV, hepatitis B virus; MVI, microvascular invasion.

Both the DFS and OS rates showed no significant differences between groups of patients stratified by clinicopathological features. The neoadjuvant RT resulted in no significant differences in DFS and OS in patients with histopathologically confirmed severe MVI (M2), mild MVI (M1) and no MVI (M0). (Fig. 3).

The impact of neoadjuvant RT was also evaluated in the subgroups of patients based on their different responses to radiation. In patients who had PR after RT, when compared with those who underwent upfront hepatectomy, neoadjuvant RT was associated with a marginally better DFS (hazard ratio, 0.19; 95% CI, 0.03–1.46) which was approaching a statistical significance (*P* = 0.076), but there was a similar OS (hazard ratio, 0.30; 95% CI, 0.04–2.31, *P* = 0.170). (Supplemental Figure A2, Supplemental Digital Content 5, http://links.lww.com/JS9/A735).

Survival analysis in the PP population which included 27 of 30 patients (89.2%) in the neoadjuvant RT group and all the 30 patients (100%) in the upfront surgery group (Fig. 1) showed the 1-year, 2-year, 3-year and 5-year DFS rates for the neoadjuvant RT group being 86.7%, 76.7%, 60.0% and 56.3%, respectively, versus the surgery upfront group being 90.0%, 66.7% 52.8% and 45.7%, respectively (*P* = 0.473). The corresponding OS rates were 96.3%, 88.9%, 85.2% and 73.3% versus 100.0%, 93.3%, 79.6% and 60.7% (*P* = 0.353). (Supplemental Figure A3, Supplemental Digital Content 6, http://links.lww.com/JS9/A736).

## Discussion

Liver resection is the established standard of care for patients with Barcelona Clinic Liver Cancer (BCLC) A stage HCC who have a sufficient reserve of liver function^4^. However, the presence of MVI in histopathological examination in resected specimens is correlated with increased incidence of recurrence after hepatectomy^15,18^. Neoadjuvant RT has been shown to be effective in improving postoperative survival in selected patients with HCC and with macrovascular invasion^8,21^. However, the present study demonstrated no significant differences in long-term DFS and OS in patients with HBV-related HCC who were predicted to have high risks of MVI after neoadjuvant RT followed by hepatectomy compared with patients after upfront hepatectomy both in the ITT or the PP analytic cohorts. Furthermore, on subgroup analyses, differences between postoperative survivals were also not significantly different in patients with histopathologically confirmed MVI using the ITT cohorts for analysis. The adverse events after neoadjuvant RT were generally mild and postoperative complications were comparable between the two groups.

Angiogenesis, being the ability of a tumour to induce new vessel formation to grow and spread, is the fundamental hallmark of cancer, including HCC^22^. MVI occurs when HCC cells have acquired a sufficient capacity to invade blood vessels, commonly portal and hepatic veins^7^. As intrahepatic dissemination of HCC cells is via the portal vein system, the vast majority of HCC recurrence after hepatectomy occurs within the liver remnant. Neoadjuvant therapies having the abilities to reduce tumour volume and destroy microscopic tumour clones can therefore reduce the possibility of tumour recurrence after liver resection for HCC. A number of studies on neoadjuvant local therapies for resectable HCC including Transarterial Chemoembolization (TACE) and Hepatic Arterial Infusion Chemotherapy (HAIC) have been reported. The impact of neoadjuvant TACE on postoperative long-term survival of HCC patients is controversial. There were one prospective randomized trial^23^ and two large retrospective cohort studies^24,25^ showing no survival benefit by using neoadjuvant TACE. On the other hand, Li *et al.* ^26^ reported a 10.5 months survival benefit for patients with a large (>10 cm) HCC tumour. Neoadjuvant HAIC before curative liver resection of HCC has been less reported. A retrospective study with a limited sample size revealed an improved event-free survival rate and OS for BCLC stage B/C HCC patients who underwent neoadjuvant HAIC (*n*=36) when compared with adjuvant portal vein-infused chemotherapy (*n*=38)^27^.

In the old days, RT resulted in suboptimal treatment results in HCC because of low radiation tolerance of liver parenchyma and inability to conform radiation doses to targeted regions^28^. With the development of precision radiation technologies which allow high doses of radiation to be delivered to the tumour while limiting radiation damage to surrounding liver parenchyma, RT has increasingly been applied in treatment of HCC either alone or in combination with other therapies. By inducing tumour necrosis and shrinkage, neoadjuvant RT may be able to eradicate or inhibit the number and extent of MVI, resulting in decrease in recurrence rate. Neoadjuvant RT has been shown in several studies in inducing HCC shrinkage with good safety^8,9,29,30^. In our previous studies on neoadjuvant RT for HCC with major portal vein tumour thrombus, the objective response rates of RT using a daily fractional dose of 3 Gy in 6 consecutive days ranged from 20.7 to 24.0%^8,9^. Only 3.7% patients encountered grade 3 adverse events after neoadjuvant RT, and similar incidences of post-hepatectomy surgical complication occurred in the 2 groups of patients with or without neoadjuvant RT. Wu and colleagues carried out a phase 2, single-arm, prospective trial to enrol 38 patients with resectable but centrally located HCC to assess the safety and impact of neoadjuvant IMRT using a treatment regimen of 50–60 Gy in 25–30 fractions over 5–6 weeks. Neoadjuvant RT resulted in a radiological response rate of 42.1% and a major pathological response rate of 34.2%. The resulted grade 3 adverse events only occurred in 3 (7.9%) patients^30^.

The present study employed the same treatment regimen as our previous studies of neoadjuvant RT for HCC with major vascular invasion^8,9^. Although a partial remission rate of 25% was reached in this study, there was no significant difference in postoperative long-term survival between the neoadjuvant RT group and the upfront surgery group. There are several possible reasons to explain why neoadjuvant RT failed to reduce recurrence rate and prolong OS: first, in the previous studies on neoadjuvant RT for HCC with portal vein tumour thrombus, the therapy was found to improve survival outcomes. This improvement was mainly due to the reduction in tumour volume of the portal vein thrombus, which is a known strong and detrimental factor of postoperative survival. The reduction in tumour thrombus volume significantly facilitated subsequent surgical treatment by increasing the rate of en-bloc resection of both the tumour and portal vein tumour thrombus, thus reducing the possibility of residual tumour or spread of tumour cells through the portal vein during surgery^8,9^. However, in this presently reported study, when compared with patients with more advanced HCC, despite the presence of MVI in the HCC patients, the reduction of tumour volume by neoadjuvant RT resulted in significantly less benefit in hepatectomy of early-stage tumours as these tumours have an extremely low possibility of residual lesion after hepatectomy even without neoadjuvant RT. Second, although RT has been shown to be effective in improving survival in HCC patients with MVI in the adjuvant settings^31,32^, the effectiveness and mechanisms involved in the neoadjuvant setting may be different. For instance, partial tumour necrosis of the main tumour induced by irradiation may cause viable tumour cells to become more likely to detach and enter into the bloodstream during hepatectomy^23^. Third, although the response to neoadjuvant RT has been found to positively correlate with more favourable outcomes after surgery, a low total dose of RT (18 Gy) was adopted in our trial to shorten the time interval between neoadjuvant treatment and surgery and to ensure safety to the subsequent hepatectomy, which yielded a relatively low response rate compared with the standard-dose radiation therapy^30,33^. Fourth, a delay in liver resection, coupled with the potential adverse effects of RT on liver parenchyma, could further compromise the surgical outcomes. Nevertheless, our data showed a concordant trend in response to RT and a better DFS approaching a marginal significance, leading to the possibility of using alternative RT regimens or combining RT with systemic drugs such as immune checkpoint inhibitors, to further improve the objective response rate of neoadjuvant therapy and postoperative long-term survival outcomes in patients with early-stage HCC.

This study has limitations. First, this is a single-institution study with a relatively small sample size, and blinding was not possible because of the nature of the intervention. Second, differences in the etiologies of HCC may lead to the different biological characteristics of tumour, thus only patients with HBV-related HCC were included in the MVI-predicating model, as well as in this study. Further research is needed to determine if the results of this study can be generalized to patients with HCC with etiologies other than HBV. Third, due to the current availability, the nomogram employed for predicting a high risk of MVI has relatively high false-positive and false-negative rates, which may limit its use in major clinical decisions. More accurate models are needed to further improve efficacy in selecting patients with a high risk of MVI to receive neoadjuvant therapies. Furthermore, the lack of variability in the ‘tumour number’ variable, due to all patients in this study having a solitary tumour, might affect the predictive performance of the nomogram for this specific population. This potential limitation should be considered when applying the nomogram in more diverse patient populations.

In conclusion, for patients with a single and small HBV-related HCC who were predicted to have high risks of MVI, although neoadjuvant RT had a promising response rate and a mild toxicity, it yielded comparable long-time disease-free and OSs after partial hepatectomy when compared with those who underwent upfront surgery.

## Ethical approval

This randomized, open-label, controlled clinical study was approved by the Ethics Committee of Eastern Hepatobiliary Surgery Hospital (EHBHKY2014-03-017), and its protocol conformed to the standards of the 1975 Declaration of Helsinki.

## Consent

Written informed consent was obtained from the patient for publication of this case report and accompanying images. A copy of the written consent is available for review by the Editor-in-Chief of this journal on request.

## Source of funding

This work was supported by the National Key R&D Program of China (No: 2022YFC2503700, 2022YFC2503705); the National Natural Science Foundation of China (No:82102941; 82103483); the San Hang Program of Navy Medical University (2021); the Project of Shanghai Municipal Science and Technology Commission (20Y11908800); Shanghai Sailing Program (20YF1459700).

## Author contribution

Concept and design: S.C., Y.Z. and W.Y.L. Patient collection and follow-up: S.C., Y.M., Y.Z., C.L., B.Z., L.H., X.W., Y.J., S.F. Data analysis: X.W., Y.J., S.F. Drafting article: X.W., Y.J., S.F. Revising article: S.C., Y.Z. and W.Y.L.

## Conflicts of interest disclosure

There are no conflicts of interest.

## Research registration unique identifying number (UIN)

The trial also conformed to the 2010 CONSORT guidelines and was registered at the Chinese Clinical Trial Registry (registration number ChiCTR-IOR-1400556; http://www.chictr.org.cn/showproj.aspx?proj=9947).

## Guarantor

Shuqun Cheng and Yaxin Zheng.

## Data availability statement

Clinical data in this study are not publicly available. The data are, however, for 3 years , available from the corresponding author (Shuqun Cheng: chengshuqun@aliyun.com), upon reasonable request and with the approval of the institutional ethical committees.

## Provenance and peer review

No.

## Supplementary Material

SUPPLEMENTARY MATERIAL

## References

[1] Sung H Ferlay J Siegel RL. Global Cancer Statistics 2020: GLOBOCAN Estimates of Incidence and Mortality Worldwide for 36 Cancers in 185 Countries. CA Cancer J Clin 2021;71:209–249. 33538338 10.3322/caac.21660

[2] Akinyemiju T Abera S Ahmed M. The Burden of Primary Liver Cancer and Underlying Etiologies From 1990 to 2015 at the Global, Regional, and National Level: results From the Global Burden of Disease Study 2015. JAMA Oncol 2017;3:1683–1691. 28983565 10.1001/jamaoncol.2017.3055 PMC5824275

[3] Llovet JM Kelley RK Villanueva A. Hepatocellular carcinoma. Nat Rev Dis Prim 2021;7:6. 33479224 10.1038/s41572-020-00240-3 PMC13537433

[4] Reig M Forner A Rimola J. BCLC strategy for prognosis prediction and treatment recommendation: The 2022 update. J Hepatol 2022;76:681–693. 34801630 10.1016/j.jhep.2021.11.018 PMC8866082

[5] Xu XF Xing H Han J. Risk factors, patterns, and outcomes of late recurrence after liver resection for hepatocellular carcinoma: a multicenter study From China. JAMA Surg 2019;154:209–217. 30422241 10.1001/jamasurg.2018.4334 PMC6439634

[6] Sumie S Kuromatsu R Okuda K. Microvascular invasion in patients with hepatocellular carcinoma and its predictable clinicopathological factors. Ann Surg Oncol 2008;15:1375–1382. 18324443 10.1245/s10434-008-9846-9

[7] Rodriguez-Peralvarez M Luong TV Andreana L. A systematic review of microvascular invasion in hepatocellular carcinoma: diagnostic and prognostic variability. Ann Surg Oncol 2013;20:325–339. 23149850 10.1245/s10434-012-2513-1

[8] Wei X Jiang Y Zhang X. Neoadjuvant three-dimensional conformal radiotherapy for resectable hepatocellular carcinoma with portal vein tumor thrombus: a randomized, open-label, multicenter controlled study. J Clin Oncol 2019;37:2141–2151. 31283409 10.1200/JCO.18.02184 PMC6698917

[9] Li N Feng S Xue J. Hepatocellular carcinoma with main portal vein tumor thrombus: a comparative study comparing hepatectomy with or without neoadjuvant radiotherapy. HPB Off J Int Hepato Pancreato Biliary Assoc 2016;18:549–556. 10.1016/j.hpb.2016.04.003 PMC4913143 27317960

[10] Moher D Hopewell S Schulz KF. CONSORT 2010 explanation and elaboration: updated guidelines for reporting parallel group randomised trials. BMJ 2010;340:c869. 20332511 10.1136/bmj.c869 PMC2844943

[11] European Association for the Study of the Liver. EASL Clinical Practice Guidelines: Management of hepatocellular carcinoma. J Hepatol 2018;69:182–236. 29628281 10.1016/j.jhep.2018.03.019

[12] Lei Z Li J Wu D. Nomogram for Preoperative estimation of microvascular invasion risk in hepatitis B virus-related hepatocellular carcinoma within the milan criteria. JAMA Surg 2016;151:356–363. 26579636 10.1001/jamasurg.2015.4257

[13] Lu XY Xi T Lau WY. Pathobiological features of small hepatocellular carcinoma: correlation between tumor size and biological behavior. J Cancer Res Clin Oncol 2011;137:567–575. 20508947 10.1007/s00432-010-0909-5 PMC11827968

[14] Zhang XP Wang K Wei XB. An Eastern Hepatobiliary Surgery Hospital microvascular invasion scoring system in predicting prognosis of patients with hepatocellular carcinoma and microvascular invasion after r0 liver resection: a large-scale, multicenter study. Oncologist 2019;24:e1476–e1488. 31138726 10.1634/theoncologist.2018-0868 PMC6975940

[15] Lim KC Chow PK Allen JC. Microvascular invasion is a better predictor of tumor recurrence and overall survival following surgical resection for hepatocellular carcinoma compared to the Milan criteria. Ann Surg 2011;254:108–113. 21527845 10.1097/SLA.0b013e31821ad884

[16] U.S. DEPARTMENT OF HEALTH AND HUMAN SERVICES: Common Terminology Criteria for Adverse Events (CTCAE) Version 5.0. 2017. https://ctep.cancer.gov/protocoldevelopment/electronic_applications/docs/ctcae_v5_quick_reference_5x7.pdf 10.3109/15360288.2015.1037530 26095483

[17] Han J Li ZL Xing H. The impact of resection margin and microvascular invasion on long-term prognosis after curative resection of hepatocellular carcinoma: a multi-institutional study. HPB Offl J Int Hepato Pancreato Biliary Assoc 2019;21:962–971. 10.1016/j.hpb.2018.11.005 30718183

[18] Roayaie S Blume IN Thung SN. A system of classifying microvascular invasion to predict outcome after resection in patients with hepatocellular carcinoma. Gastroenterology 2009;137:850–855. 19524573 10.1053/j.gastro.2009.06.003 PMC2739450

[19] Sumie S Nakashima O Okuda K. The significance of classifying microvascular invasion in patients with hepatocellular carcinoma. Ann Surg Oncol 2014;21:1002–1009. 24254204 10.1245/s10434-013-3376-9

[20] Clavien PA Barkun J de Oliveira ML. The Clavien-Dindo classification of surgical complications: five-year experience. Ann Surg 2009;250:187–196. 19638912 10.1097/SLA.0b013e3181b13ca2

[21] Wei XB Xu J Li N. The role of three-dimensional imaging in optimizing diagnosis, classification and surgical treatment of hepatocellular carcinoma with portal vein tumor thrombus. HPB Off J Int Hepato Pancreato Biliary Assoc 2016;18:287–295. 10.1016/j.hpb.2015.10.007 PMC4814596 27017169

[22] Hanahan D. Hallmarks of cancer: new dimensions. Cancer Discov 2022;12:31–46. 35022204 10.1158/2159-8290.CD-21-1059

[23] Zhou WP Lai EC Li AJ. A prospective, randomized, controlled trial of preoperative transarterial chemoembolization for resectable large hepatocellular carcinoma. Ann Surg 2009;249:195–202. 19212170 10.1097/SLA.0b013e3181961c16

[24] Lee KT Lu YW Wang SN. The effect of preoperative transarterial chemoembolization of resectable hepatocellular carcinoma on clinical and economic outcomes. J Surg Oncol 2009;99:343–350. 19226530 10.1002/jso.21248

[25] Shi HY Wang SN Wang SC. Preoperative transarterial chemoembolization and resection for hepatocellular carcinoma: a nationwide Taiwan database analysis of long-term outcome predictors. J Surg Oncol 2014;109:487–493. 24293372 10.1002/jso.23521

[26] Li C Wang MD Lu L. Preoperative transcatheter arterial chemoembolization for surgical resection of huge hepatocellular carcinoma (>/= 10 cm): a multicenter propensity matching analysis. Hepatol Int 2019;13:736–747. 31486964 10.1007/s12072-019-09981-0

[27] Pan Y Mei J Chen J. Comparison between portal vein perfusion chemotherapy and neoadjuvant hepatic arterial infusion chemotherapy for resectable intermediate to advanced stage hepatocellular carcinoma. Ann Surg Oncol 2022;29:2016–2029. 34637058 10.1245/s10434-021-10903-4

[28] Ohri N Dawson LA Krishnan S. Radiotherapy for hepatocellular carcinoma: new indications and directions for future study. J Natl Cancer Inst 2016;108:djw133. 27377923 10.1093/jnci/djw133 PMC6279296

[29] Wei Z Zhao J Bi X. Neoadjuvant radiotherapy for resectable hepatocellular carcinoma with portal vein tumor thrombus: a systematic review. Hepatobil Surg Nutr 2022;11:709–717. 10.21037/hbsn-20-854 PMC9577988 36268237

[30] Wu F Chen B Dong D. Phase 2 evaluation of neoadjuvant intensity-modulated radiotherapy in centrally located hepatocellular carcinoma: a nonrandomized controlled trial. JAMA Surg 2022;157:1089–1096. 36197682 10.1001/jamasurg.2022.4702 PMC9535533

[31] Shi C Li Y Geng L. Adjuvant stereotactic body radiotherapy after marginal resection for hepatocellular carcinoma with microvascular invasion: a randomised controlled trial. Eur J Cancer 2022;166:176–184. 35303509 10.1016/j.ejca.2022.02.012

[32] Wang L Wang W Yao X. Postoperative adjuvant radiotherapy is associated with improved survival in hepatocellular carcinoma with microvascular invasion. Oncotarget 2017;8:79971–79981. 29108379 10.18632/oncotarget.20402 PMC5668112

[33] Chen CP. Role of external beam radiotherapy in hepatocellular carcinoma. Clin Liver Dis 2020;24:701–717. 33012454 10.1016/j.cld.2020.07.006

