# Supplementary material for: Neoadjuvant intensity modulated radiotherapy for a single and small (≤5 cm) hepatitis B virus-related hepatocellular carcinoma predicted to have high risks of microvascular invasion: a randomized clinical trial
Source: Int J Surg. 2023 Jun 22;109(10):3052–60. doi: 10.1097/JS9.0000000000000574 (PMC10583963; doi:10.1097/JS9.0000000000000574)
Supplement: SUPPLEMENTARY MATERIAL [file js9-109-3052-s002.docx]

**Appendix**

**Resectability of tumor**

The resectability of tumor in patients with hepatocellular carcinoma was determined by experienced surgeons. Patients were not considered for surgery if they had one or more of the following clinical features: Eastern Cooperative Oncology Group performance status > 2; multiple disseminated nodules (>3) on imaging examinations; distant metastasis; Child-Pugh class C liver function; Child-Pugh class B liver function for a tumor which required major hepatectomy which was defined as resection of three or more Couinaud liver segments; invasion of major portal or hepatic vein and severe co-existing systemic diseases.

**Surgical Procedure**

(1) The conventional open approach is used in this study;

(2) A right subcostal incision is recommended, which will be adjusted according to the patient’s condition to facilitate surgery;

(3) Abdominal cavity exploration is performed to exclude possible distant metastasis or other pathological conditions;

(4) Liver exploration is carried out to determine the size, number and location of the tumor, satellite nodules, cirrhosis, and re-determine the tumor resectability and the volume of future liver remnant;

(5) Intraoperative ultrasound (IUS) is routinely used to identify any additional small nodules not detected on preoperative imaging studies or at liver exploration, and to confirm the relationships between the tumors and intrahepatic vascular and biliary structures;

(6) The operation is performed with an intention of complete removal of all macroscopic tumor nodules and obtaining a resection margin of ≥ 1.0 cm. The extent (major vs. minor) and type (anatomic vs. non-anatomic) of hepatectomy will be determined by the operating surgeons based on the size, number and location of recurrent tumors, degree of cirrhosis, resection margin and the estimated volume of future liver remnant;

(7) Pringle’s maneuver or selective hepatic vascular exclusion is allowed. Blood inflow is blocked only when there is more bleeding in the hepatic cutting surface. A clamp/unclamp time of 15 minutes/5 minutes policy is used if Pringle’s maneuver is performed;

(8) Techniques of liver parenchymal transection including finger fracture technique, clamp-crush method and Cavitron ultrasonic surgical aspirator (CUSA) are allowed to use;

(9) A proper and thorough hemostasis should be achieved in the hepatic cutting surface. The cutting surface can be stitched, or coated with medical glue and hemostatic gauze, or managed with other methods which can be determined by the operating surgeons; abdominal drainage is routinely performed;

(10) Other intraoperative anti-cancer treatments such as hepatic artery ligation, intra-arterial drug infusion and/or embolization, and brachytherapy should not be used;

**Follow-up protocol**

The patients were followed-up in the outpatient clinic once a month in the first year, and subsequently at longer intervals. Serum alpha-fetoprotein measurements and abdominal ultrasounds were performed once every month. Contrast CT or MRI was performed once every three months for surveillance of recurrence, or if HCC recurrence was suspected clinically. TACE, local ablative therapy, or systemic therapy was used for the treatment of HCC recurrence, depending on the location of the recurrent lesion, size and number of recurrence, liver functional status, and presence/absence of extrahepatic disease. Palliative treatment was provided to patients with an end-stage disease, poor general status or poor liver function.
