# Supplementary figures and images for: Neoadjuvant intensity modulated radiotherapy for a single and small (≤5 cm) hepatitis B virus-related hepatocellular carcinoma predicted to have high risks of microvascular invasion: a randomized clinical trial
Source: Int J Surg. 2023 Jun 22;109(10):3052–60. doi: 10.1097/JS9.0000000000000574 (PMC10583963; doi:10.1097/JS9.0000000000000574)

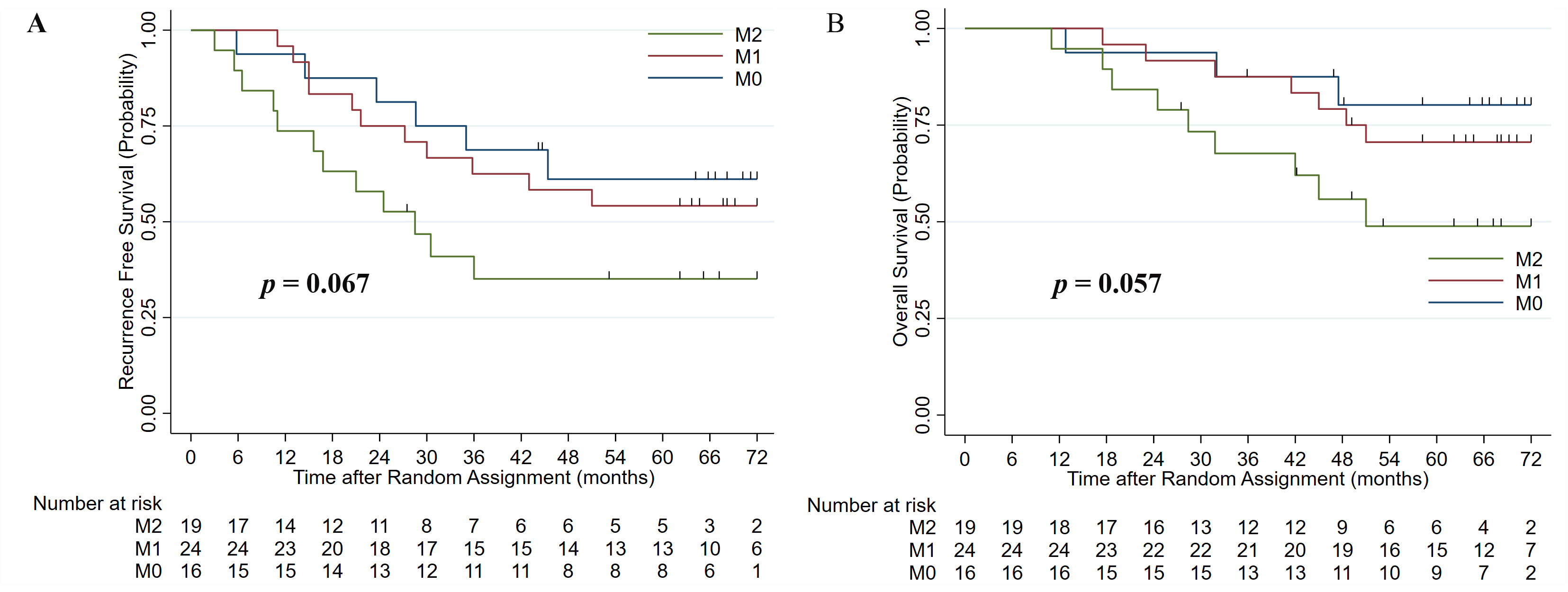

Supplement: SUPPLEMENTARY MATERIAL [file js9-109-3052-s003.jpg]

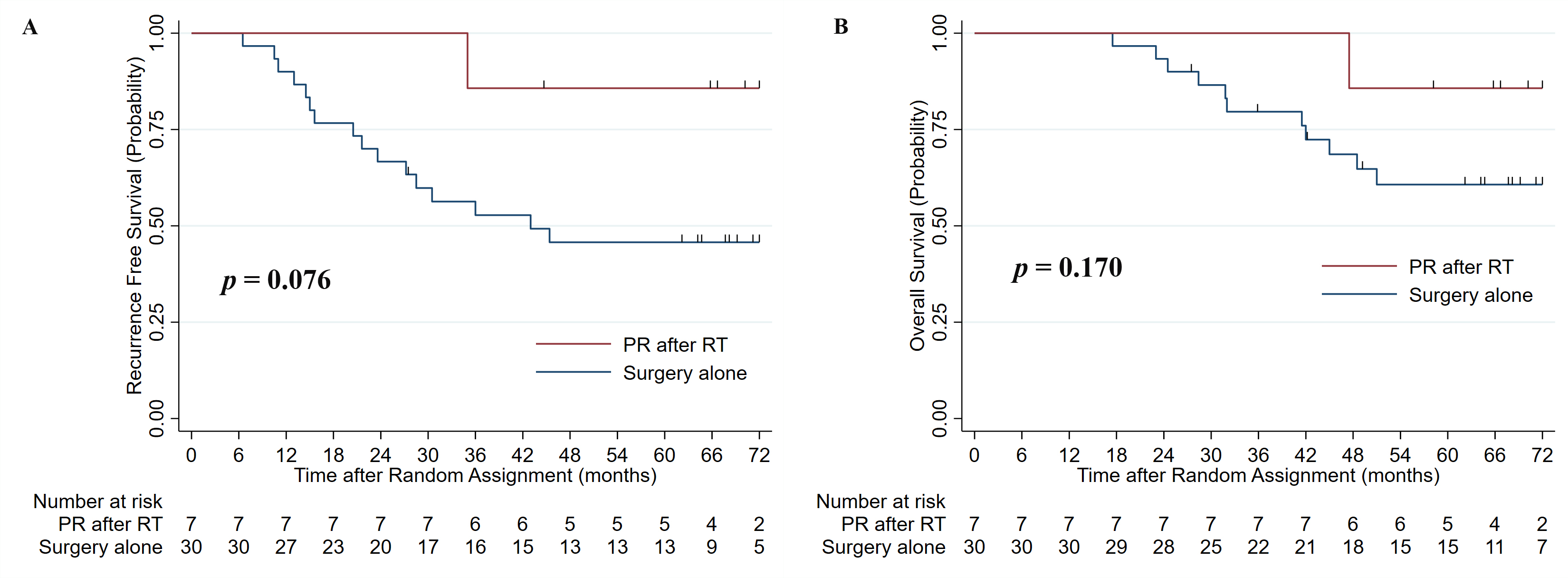

Supplement: SUPPLEMENTARY MATERIAL [file js9-109-3052-s005.jpg]

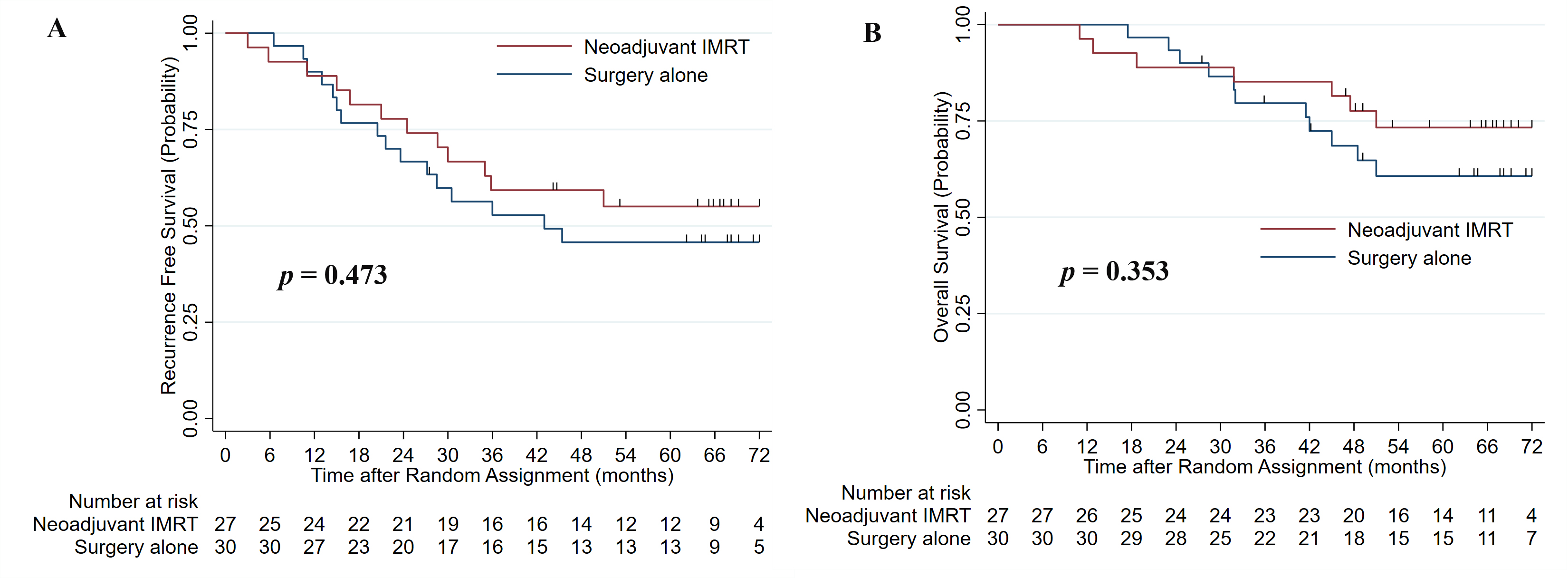

Supplement: SUPPLEMENTARY MATERIAL [file js9-109-3052-s006.jpg]
