# Supplementary material for: Neoadjuvant intensity modulated radiotherapy for a single and small (≤5 cm) hepatitis B virus-related hepatocellular carcinoma predicted to have high risks of microvascular invasion: a randomized clinical trial
Source: Int J Surg. 2023 Jun 22;109(10):3052–60. doi: 10.1097/JS9.0000000000000574 (PMC10583963; doi:10.1097/JS9.0000000000000574)
Supplement: SUPPLEMENTARY MATERIAL [file js9-109-3052-s004.docx]

**Supplemental Table A1: The detailed information of recurrences**

|  | Recurrences in neoadjuvant RT group  ( n = 13 ) | Recurrences in Surgery alone group  ( n= 16 ) | *p* value |
| --- | --- | --- | --- |
| Time interval |  |  |  |
| Early recurrence (<= 2 years) | 7 | 10 | 0.567 |
| Late recurrence (> 2 years) | 6 | 6 | 1.0 |
| Patterns of recurrence |  |  |  |
| Single intra-hepatic recurrence | 6 | 14 | 0.041^*^ |
| Multiple intra-hepatic recurrence | 6 | 2 |  |
| Extrahepatic metastasis | 1 | 0 |  |
| Sites of intra-hepatic recurrence |  |  |  |
| Resected margin | 2 | 4 | 0.798 |
| Confined in the ipsilateral hemiliver | 5 | 6 |  |
| Involved the contralateral hemiliver | 6 | 6 |  |

The number of patients had HCC recurrence were 13 in neoadjuvant RT group and 16 in surgery alone group.

RT: radiotherapy

**Supplemental Table A2. The initial salvage therapies for recurrences**

| Treatments | Recurrences in neoadjuvant RT group ( n = 13 ) | Recurrences in Surgery alone group ( n = 16 ) | *p* |
| --- | --- | --- | --- |
| Re-operation | 6 | 11 | 0.274 |
| TACE | 3 | 2 | 0.632 |
| PRFA | 1 | 2 | 1.0 |
| RT | 1 | 0 | 0.448 |
| Systmeic drugs | 2 | 1 | 0.573 |

TACE: transcatheter arterial chemoembolization; PRFA: percutaneous radiofrequency ablation; RT: radiotherapy
